# Changes in Large Pulmonary Arterial Viscoelasticity in Chronic Pulmonary Hypertension

**DOI:** 10.1371/journal.pone.0078569

**Published:** 2013-11-06

**Authors:** Zhijie Wang, Roderic S. Lakes, Mark Golob, Jens C. Eickhoff, Naomi C. Chesler

**Affiliations:** 1 Department of Biomedical Engineering, University of Wisconsin – Madison, Madison, Wisconsin, United States of America; 2 Department of Engineering Physics, University of Wisconsin – Madison, Madison, Wisconsin, United States of America; 3 Department of Material Science, University of Wisconsin – Madison, Madison, Wisconsin, United States of America; 4 Department of Biostatistics and Medical Informatics, University of Wisconsin – Madison, Madison, Wisconsin, United States of America; Emory University/Georgia Insititute of Technology, United States of America

## Abstract

Conduit pulmonary artery (PA) stiffening is characteristic of pulmonary arterial hypertension (PAH) and is an excellent predictor of mortality due to right ventricular (RV) overload. To better understand the impact of conduit PA stiffening on RV afterload, it is critical to examine the arterial viscoelastic properties, which require measurements of elasticity (energy storage behavior) and viscosity (energy dissipation behavior). Here we hypothesize that PAH leads to frequency-dependent changes in arterial stiffness (related to elasticity) and damping ratio (related to viscosity) in large PAs. To test our hypothesis, PAH was induced by the combination of chronic hypoxia and an antiangiogenic compound (SU5416) treatment in mice. Static and sinusoidal pressure-inflation tests were performed on isolated conduit PAs at various frequencies (0.01–20 Hz) to obtain the mechanical properties in the absence of smooth muscle contraction. Static mechanical tests showed significant stiffening of large PAs with PAH, as expected. In dynamic mechanical tests, structural stiffness (κ) increased and damping ratio (D) decreased at a physiologically relevant frequency (10 Hz) in hypertensive PAs. The dynamic elastic modulus (E), a material stiffness, did not increase significantly with PAH. All dynamic mechanical properties were strong functions of frequency. In particular, κ, E and D increased with increasing frequency in control PAs. While this behavior remained for D in hypertensive PAs, it reversed for κ and E. Since these novel dynamic mechanical property changes were found in the absence of changes in smooth muscle cell content or contraction, changes in collagen and proteoglycans and their interactions are likely critical to arterial viscoelasticity in a way that has not been previously described. The impact of these changes in PA viscoelasticity on RV afterload in PAH awaits further investigation.

## Introduction

Pulmonary arterial hypertension (PAH) is the most severe form of pulmonary hypertension (PH), with a mortality rate of approximately 15% within 1 year on modern therapy [1]. PAH is characterized by vascular remodeling in pulmonary arteries (PAs) including extracellular matrix (ECM) deposition, intimal thickening and smooth muscle cell (SMC) hypertrophy and proliferation. However, the cause of death in PAH is typically right heart failure, which implies an important interaction between the vascular changes and ventricular dysfunction.

Progressive right ventricular (RV) dysfunction, which is a precursor to RV failure, occurs during PH as a consequence of increased RV afterload that consists of the steady and oscillatory loads that oppose the ejection of blood during ventricular systole. RV dysfunction is traditionally thought to be due to progressive distal PA narrowing, which contributes only to the steady component of the RV afterload and is not a good predictor of mortality in PAH [2], [3]. The oscillatory component of the RV afterload is affected by pulmonary vascular compliance, which may be why proximal, conduit PA stiffening is a strong predictor of mortality in PAH [4]–[9]. To understand how and to what extent proximal PA stiffening contributes to RV failure, the first step is to comprehensively investigate the biomechanical changes of the proximal PAs caused by PAH.

It is well known that arteries behave as viscoelastic materials in the dynamic, pulsatile blood flow environment; a characteristic of viscoelastic materials is frequency dependent behavior [10]–[13]. Arterial elasticity describes how much energy is stored (maintained) in each oscillatory deformation, whereas arterial viscosity reflects how much energy is dissipated (lost). Together, viscoelastic properties enable arteries to serve as both conduits and buffers for the pulsatile blood flow generated by the contracting heart. Thus, viscoelastic properties have profound influences on blood flow, energy dissipation and even ventricular function [14]–[18].

Most prior studies on large artery viscoelasticity have focused on the effect of SMC content and contractile state; however, little is known about how ECM protein accumulation, which often occurs in pathological remodeling such as due to hypertension, alters arterial viscoelasticity. Proximal PA stiffening is universally observed in pulmonary hypertension and is strongly associated with changes in ECM [19]–[23]. In particular, our group has shown that collagen accumulation is mainly responsible for proximal PA stiffening due to hypoxic pulmonary hypertension (HPH) in mice and that PA viscoelasticity is altered even when SMC content and contractile state are unchanged [24], [25]. These results suggest that SMCs are not the only source of viscoelastic properties in arteries and that ECM proteins such as collagen may play an important role.

Here we sought to comprehensively investigate the proximal PA viscoelastic property changes caused by PAH and the likely biological factors responsible. We previously demonstrated that large PA viscoelastic properties, measured at a single, low testing frequency (0.01 Hz), are altered by chronic hypoxia^19^, which is a common way of creating mild PAH experimentally, especially in rodents[26], [27]. However, an important knowledge gap is how the viscoelastic behavior in a wide range of frequencies is altered in large PAs (especially frequencies in a physiological range), by a degree of PAH that is more clinically relevant (i.e., severe PAH). We hypothesized that severe PAH leads to frequency-dependent changes in arterial stiffness and damping capacity in conduit PAs, and that these properties are correlated with ECM accumulation. To test our hypotheses, we used a new murine model of severe PAH [28] and performed in vitro mechanical tests on the extralobar PAs from hypertensive and control mice to obtain the viscoelastic behavior at various testing frequencies. Biological changes in these PAs were then examined to identify factors responsible for the viscoelastic changes.

## Methods

All procedures were approved by the University of Wisconsin–Madison Institutional Animal Care and Use Committee. Twelve 10–12 week old, male C57BL/6 mice obtained from Jackson Laboratory (Bar Harbor, ME) were randomized to normoxia exposure (N = 6) as controls or 21 days of hypoxia with single weekly SUGEN treatment (SU5416; Sigma-Aldrich Corp., intraperitoneal injection) at the dose of 20 mg/kg[28] (N = 6) to induce severe PAH (21d HySu group). SUGEN, the vascular endothelial growth factor (VEGF) receptor blocker, was suspended in CMC (0.5% [w/v] carboxymethyl cellulose sodium, 0.9% [w/v] sodium chloride, 0.4% [v/v] polysorbate 80, 0.9% [v/v] benzyl alcohol in deionized water). During hypoxia, animals were maintained in a normobaric hypoxic chamber at a controlled O_2_ concentration of 10% with 4 L min^−1^ air flow to maintain the carbon dioxide level at < 600 ppm[24]. The chambers were opened for less than 30 minutes at a time weekly for regular animal care or SU5416 injections. Control mice were housed in normal room air. We did not include the control group with SUGEN treatment alone because previous studies using this rodent HySu model have shown minimal effects of SUGEN on severe PAH progression[28], [29].

### Static and dynamic mechanical tests

At the end of the exposure period, mice were euthanized with an overdose of 50 mg/ml pentobarbital by intraperitoneal injection. Then a midline sternotomy was performed, and the heart and lungs were removed. Extralobar PAs were excised from the pulmonary trunk to the first branches under microscopy. Left PAs were mounted to aligned cannulas in a vessel chamber (Living System Instrumentation (LSI), Burlington, VT) with a no-flow setup in which the vessel was pressurized by symmetric inflow to each cannula for static and dynamic mechanical testing. Two pressure transducers were placed a short distance away from each cannula to obtain pressures at both ends of the vessel. We confirmed the negligible phase difference between the pressures measured at each cannula, which indicates that there was minimal viscoelastic effect of the fluid system on the results obtained. After the mechanical tests, left PAs were fixed in 10% formalin for histology analysis (within 8 h of euthanasia).

In the testing system, PAs were stretched axially 140%, at the approximate in vivo stretch ratio, to prevent buckling at higher pressures and tested at this fixed length as done in prior studies [20], [24], [30]. Because SMC tone is generally minimal in proximal PAs, even with HPH [31], and because we were interested in ECM-regulated viscoelastic behavior, mechanical testing was performed in the absence of SMC tone (i.e., in a passive state) by using calcium- and magnesium-free PBS for both perfusate and superfusate. The perfusate was supplied via a steady flow pump (LSI; Burlington, VT) and an oscillatory flow pump (EnduraTec TestBench; Bose Corporation; Eden Prairie, MN) to achieve static pressurization or cyclic sinusoidal pressurization from 10 to 50 mmHg at frequencies of 0.01, 0.1, 1, 3, 5, 7, 10, 15 and 20 Hz. Superfusate was continuously circulated and maintained at 37°C. Between the dynamic and static mechanical tests, the vessels were allowed 30 minutes to equilibrate, then preconditioned at 0.014 Hz for 10 cycles before data recording as done previously [20].

In order to quantify viscoelasticity at physiologically relevant frequencies, we replaced the pressure transducers and amplifiers used in our previous isolated vessel test system (PT-F and PM-4, LSI) with ones with higher frequency response but similar precision. We then characterized the frequency response of gain and phase delay of these pressure transducers (APT300, Harvard Apparatus) and amplifiers (HSE TAM-A, Harvard Apparatus). The systemic error at the frequency of 20 Hz was 1% in phase delay and 0% in gain. In addition, to examine whether our testing system would induce resonance at the testing frequencies which would interfere with the viscoelasticity measurement, we tested lab-made silicone discs in a separate viscoelastic testing system [32], [33] and lab-made silicone tubes in our vessel testing system. The material used for the discs and the tubes were identical and the forming techniques were similar. Also, the silicone tubes had similar OD and wall thickness as the mouse arteries. As measured in the disc experiments, the silicone material resonance frequency varied from ∼400 to ∼900 Hz. The damping capacity measured from disc and tube experiments was constant within the narrower testing frequency range of the tube experiments (up to 20 Hz) and was consistent between the two types of experiments (data not shown). These results provide additional validation of the modified test system for the frequency range of testing performed here.

Pressure-diameter data were recorded simultaneously (IonWizard software Version 6.0, IonOptix, Milton, MA) using in-line pressure transducers at the acquisition frequency of 1 Hz (for static testing and dynamic testing at the frequency of 0.01 Hz) or 250 Hz (for dynamic tests at all other testing frequencies) and a CCD camera (IonOptix, Milton, MA) connected to an inverted microscope (Olympus, Center Valley, PA) to measure OD at 4× magnification with the acquisition frequency of 1.2 Hz (for static testing and dynamic testing at the frequency of 0.01 Hz) or 240 Hz (for dynamic tests at all other testing frequencies). During the static test, pressure stair steps of 5, 10, 15, 20, 25, 30, 35, 40, 45 and 50 mmHg were applied for 30 seconds each and pressure- diameter data were recorded as done in the dynamic test. Finally, vessel plastic deformation was checked by OD at a final 5 mmHg step and no significant change in OD after the mechanical tests was observed.

### Analysis of vessel mechanical properties

The viscoelastic and static mechanical properties of PAs were obtained from pressure-stretch as well as stress-strain relationships. Stretch (λ) was calculated as the ratio of pressure-dependent OD to OD at the baseline pressure of approximately 0 mmHg (OD_0_) for dynamic and static mechanical tests. The arteries were assumed to be homogeneous and incompressible. Assuming conservation of mass and no axial extension, the wall thickness (h) as a function of OD at a given pressure was calculated as:
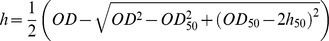



where OD_50_ and h_50_ are the OD and h measured optically at 50 mmHg with the same inverted microscope and CCD camera described above. Then, we calculated the Piola-Kirchhoff circumferential stress (S)[34] by 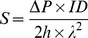



where P is the transmural pressure and ID is the inner diameter. Green circumferential strain (ε) was calculated as 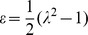



Then we calculated the elastic modulus (E) as the slope of the entire stress-strain (S-ε) curve for the static tests or the slope of the line best fit to the loading S-ε curve for the dynamic tests. For the static tests, the strain at which the S-ε curve transitioned to a steeper slope (i.e., the transition strain) was calculated as described previously[25].

From the dynamic tests, the dynamic structural stiffness (κ) was defined by the slope of the line best-fit to the loading cycle of pressure-stretch hysteresis loop during the 10–50 mmHg cyclic sinusoidal pressurization.

Note that static E, dynamic E and dynamic κare single, linearized metrics of nonlinear, viscoelastic behavior. Therefore, differences in stiffness and elastic modulus that are apparent as a function of strain and loading vs. unloading (in the dynamic tests) are not captured. For a more comprehensive assessment, full stress-strain curves and hysteresis loops are required.

Next, a viscous property of the PA, which describes the ratio of energy loss to total energy in the artery wall over a pressurization cycle, was obtained as follows: the stored energy (W_S_) and dissipated energy (W_D_) were calculated as area of the triangle defined by the maximum and minimum stretch in the hysteresis loop and the area within the hysteresis loop, respectively. Then, damping capacity (D) was calculated as W_D_/(W_D_ + W_S_). These methods have been described previously[25]. Damping capacity was also calculated from the dynamic S-ε hysteresis loops in the same way.

### Histology and Immunohistochemistry

After mechanical tests, left PAs were saved for histology by fixation at zero transmural pressure in 10% formalin and preservation in 70% ethanol. The vessels were then embedded in agar gel, sectioned, and stained with hematoxylin and eosin (H&E) to measure overall morphology and geometry, with Verhoff Van Geisen (VVG) to identify elastin and with picro-sirius red to identify collagen. Furthermore, the sections were stained with alcian blue to identify all proteoglycans. Sections were imaged on an inverted microscope (TE-2000; Nikon, Melville, NY) and captured using a Spot camera and software for image capture and analysis (Meta Vue; Optical Analysis Systems, Nashua, NH). To avoid the artifact induced by fixation or sectioning, only ‘intact’ regions (e.g., minimal gaps between arterial layers) were chosen for the histology quantification of each artery. To quantify the collagen or proteoglycans in the PAs, the area positive for collagen or proteoglycans (i.e.,red or blue color) was identified by color thresholding in the fields of view by a single observer blinded to the experimental condition. Total collagen or proteoglycans were then measured as the product of area fraction and total wall thickness of the PAs as done previously [20].

To measure the cell proliferation and apoptosis in large PAs with severe PAH, we performed immunohistochemistry using rabbit polyclonal to PCNA (Abcam, ab2426) and Caspase 3 (Abcam, ab52294) antibodies, respectively. The secondary antibody used was goat polyclonal antibody to rabbit IgG (Abcam, ab6012). All slides were counterstained with hematoxylin. To quantify the proliferation and apoptosis rates, the numbers of positively stained cells and all cells in an arbitrarily selected area were counted using ImageJ (NIH) by a single observer blinded to the experimental condition. Then the percentage of positively stained cells was calculated.

### Hemodynamic measurements

In a separate group of animals subject to the same experimental treatments (control and 21d HySu), mice were anesthetized with urethane and open chest surgery was performed. Next, in vivo RV pressure was measured by right heart catheterization using a 1.2 F admittance pressure-volume catheter (Scisense, Ithaca, NY). The RV pressure was continuously recorded at 1,000 Hz on commercially available software (Notocord Systems, Croissy Sur Seine, France). The detailed method has been described previously[35]. At the end of experiments, mice were euthanized by cervical dislocation. The weights of RV free wall, LV and septum were measured and Fulton Index was then calculated as RV/(LV+S) to measure RV hypertrophy.

### Statistics

All results are presented as mean ± SE. Analysis of variance with repeated measurements and a compound symmetry structure was used to compare all outcome parameters between the 21d HySu group and the control group. The rationale for conducting the analysis based on the compound symmetry assumption was the small sample size (5–6 mice per group), which prevented us from utilizing a more flexible correlation structure in the linear mixed effects model analysis. All P-values were two-sided and P<0.05 was used to define statistically significance.

## Results

### Development of severe pulmonary arterial hypertension

**Figure 1:**
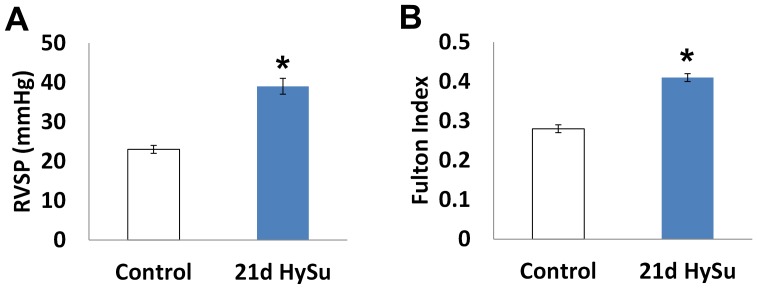
Right ventricular systolic pressure (RVSP) and Fulton index (RV/(LV+S)) measured from control and 21d HySu groups. N  =  5–9 per group. Means ± Standard Errors. * p<0.05 vs. control.

The HySu mouse model of severe PAH is recently established[28] and we employed this model to study the vascular changes that occur with severe PAH. To confirm the development of severe PAH, we measured the right ventricular systolic pressure (RVSP) by right heart catheterization and right ventricular hypertrophy by the Fulton Index. As shown in Fig 1, we observed a significant increase in RVSP and development of RV hypertrophy in the 21d HySu group (p<0.05). While the degree of hypertension is not as high as previously measured using a closed chest technique (>45 mmHg) [28], we can categorize it as severe based on the Fulton index (>0.35) [28]. Moreover, the RVSP in the 21d HySu group was significantly higher than the peak systolic PA pressure of mice exposed to hypoxia alone measured using the same open chest technique (39±2 vs. 31±2 mmHg; p<0.05) [36].

### Geometrical and biological changes in PA

From our isolated vessel tests, we found the OD of large PAs measured optically at 50 mmHg was reduced by severe PAH (1179±26 µm in control vs. 880±26 µm in 21d HySu, p<0.05) while wall thickness measured by histology was increased (35±3 µm in control vs. 49±4 µm in 21d HySu, p<0.05).

**Figure 2:**
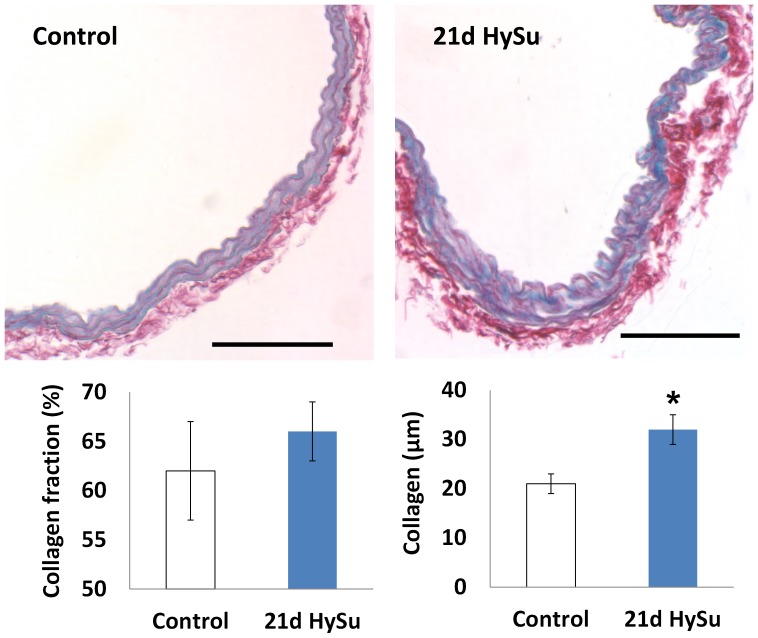
Sirius red staining for collagen (red) in control and 21d HySu PAs. Scale bar  =  100 µm. Collagen fraction area (%) tended to increase and total collagen thickness was increased in 21d HySu PAs. N  =  6 per group. Means ± Standard Errors * p<0.05 vs. control.

**Figure 3:**
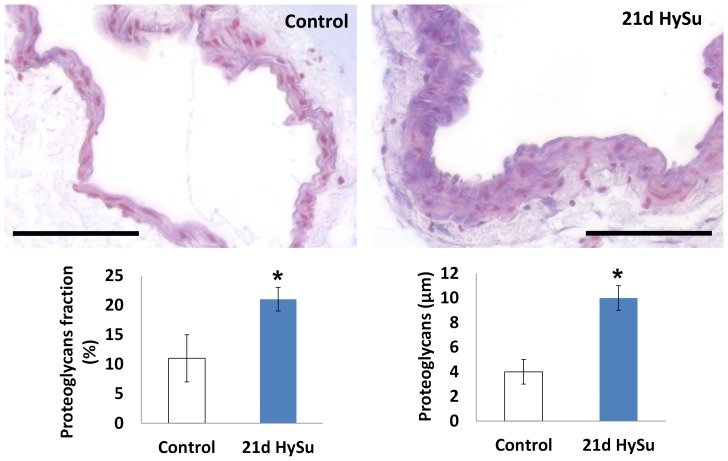
Alcian blue staining for proteoglycans (blue) in control and 21d HySu PAs. Scale bar  =  100 µm. Both area fraction (%) and the total thickness of proteoglycans were increased in 21d HySu PAs. N  =  6 per group. Means ± Standard Errors. * p<0.05 vs. control.

We also found an increase in collagen in the large PAs with severe PAH (Fig 2) as expected but did not observe a significant change in elastin (results not shown). Moreover, proteoglycan content increased significantly (Fig 3). Immunohistochemical assays for cell proliferation and apoptosis showed no significant changes with PAH. For proliferation: 55±27% in 21d HySu vs. 41±14% in control, p = 0.29; for apoptosis: 59±21% in 21d HySu vs. 47±19% in control, p = 0.35.

### Static mechanical properties

**Figure 4:**
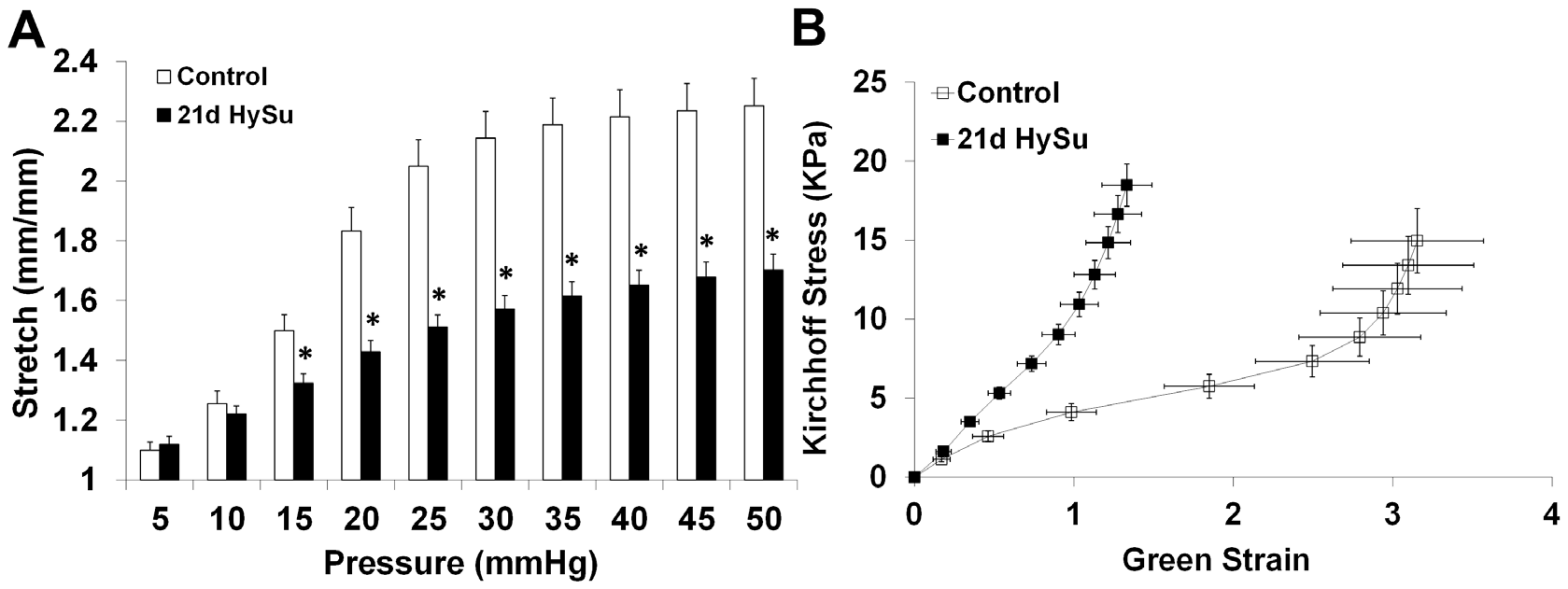
Pressure-stretch (A) and Kirchhoff stress- Green strain (B) relationships of large PAs obtained from static mechanical tests. N  =  5 per group. Means ± Standard Errors. * p<0.05 vs. control.

The pressure-stretch and stress-strain relationships were examined to quantify static mechanical behavior. In the 21d HySu group, stretch was significantly reduced by 12 – 27% at inflating pressures of 15 – 50 mmHg, as shown in Fig 4A, indicating static structural stiffening. We further examined the stress-strain curves and found a leftward shift of the curve in the 21d HySu group compared to the control group (Fig 4B). Transition strain was decreased by 68%, and the linearized measure of elastic modulus over the entire testing range E was increased by 217% with 21d HySu (4.4±1.3 kPa vs. 14.0±2.4 kPa, p<0.05). These changes in static mechanical properties indicate marked static structural and material stiffening of large PAs due to PAH.

### Dynamic mechanical properties

**Figure 5:**
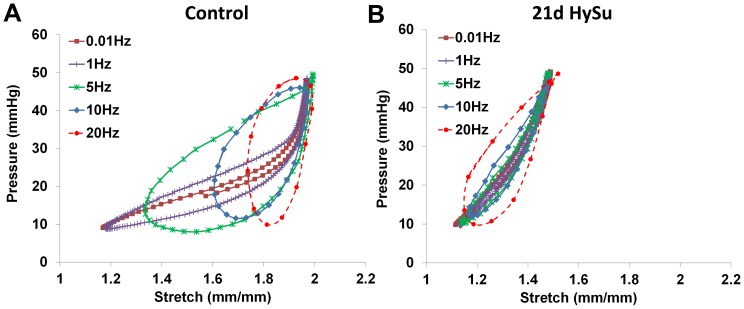
Examples of pressure-stretch curves obtained from control (A) and 21d HySu (B) PAs.

From the dynamic mechanical tests, we found very different behavior between the 21d HySu group and the control group. The changes are illustrated in Fig 5. In the control PAs, as the testing frequency increased, the minimal stretch point of the loop increased whereas the maximal stretch point remained the same. As a result, the loop shape became rounder with the stretch range narrowing as the testing frequency increased (Fig 5A). However, in the hypertensive PAs, this frequency-dependent behavior disappeared;both the minimal and maximal stretch points remained similar over the testing frequency range (Fig 5B). In both control and hypertensive PAs, overall loop area increases were evident as the frequency increased.

With quantification of the hysteresis loops, we observed PAH- and frequency-dependent changes in the three parameters that quantify dynamic structural stiffness, dynamic material stiffness and damping capacity – κ, E and D - between the 21d HySu and control groups.

**Figure 6:**
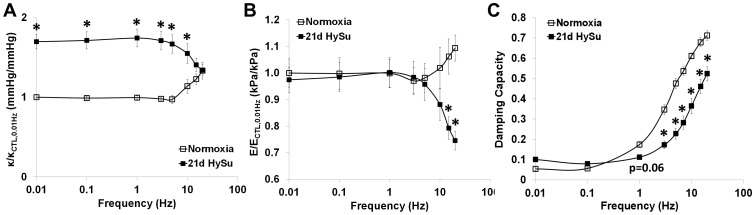
Frequency-dependent dynamic structural stiffness κ (A), dynamic elastic modulus E, (B) and damping capacity D (C) in control and 21d HySu PAs. N  =  5 per group. Means ± Standard Errors. * p<0.05 vs. control.

In Figure 6A, we present the results in the form of κ/κ_CTL, 0.01 Hz_, i.e., κ normalized by that of control PAs at a quasi-static frequency of 0.01 Hz. In control PAs, we observed an initial plateau of this normalized κ at frequencies lower than 1 Hz and then a near-exponential increase with increasing testing frequency. Severe PAH led to an overall increase in κ at 0.01–10 Hz (p<0.05) and a similar initial plateau of the normalized κ at frequencies lower than 1 Hz; however, as the frequency further increased, κ decreased (Fig 6A).

The dynamic material stiffness of the PAs quantified by the dynamic elastic modulus (E) also showed frequency-dependent differences between the 21d HySu and control groups. As shown in Fig 6B, there was no difference in E/E_ CTL, 0.01 Hz_ between control and hypertensive PAs at 0.01–10 Hz. Similar to κ/κ_CTL, 0.01 Hz_, as the frequency increased, the normalized E increased in control PAs whereas it decreased in the 21d HySu group. As a result, at 15 and 20 Hz, E/E_ CTL, 0.01 Hz_ was significantly lower in the hypertensive PAs compared to the control PAs (p<0.05).

Interestingly, the linearized dynamic elastic modulus was generally greater than the linearized static elastic modulus and did not increase with severe PAH (∼60 kPa at 0.01 Hz for both groups compared to ∼4 kPa for control PAs and ∼14 kPa for hypertensive PAs measured statically).

In terms of damping capacity D (Fig 6C), both control and hypertensive PAs showed an increase with increasing testing frequency for frequencies above 1 Hz. Moreover, at the higher frequencies (>1 Hz), D was significantly reduced in the 21d HySu group compared to the control group (p<0.05).

## Discussion

In the present study, frequency-dependent viscoelastic properties were measured in extralobar PAs of control mice and those with severe PAH for the first time. In control mice, the structural stiffness (κ), elastic modulus (E), and damping capacity (D) were frequency-dependent in a physiologically-relevant range of frequencies. In particular, κ, E, and D all increased with increasing frequency above 1 Hz. This type of behavior remained for D but reversed with κ and E in mice with severe PAH. Moreover, compared to controls, there was an increase in κ and decrease in D at the normal mouse heart rate (∼10 Hz) with severe PAH. The dynamic material stiffness (E) did not increase with severe PAH, which is in contrast to the static material stiffness. The measured increases in collagen and proteoglycan were likely responsible for most of these mechanical changes since SMCs were inactive and the amount did not change with severe PAH. Thus, our results suggest that ECM proteins and their dynamic interactions play an important role in arterial viscoelasticity changes with PAH.

In the present PAH model with VEGF receptor inhibitor (SU5416), vascular remodeling is likely associated with endothelial cell dysfunction in distal, small PAs [37], [38]. As a result, the model is favored because animals develop plexiform-like lesions, dramatic increase in mean pulmonary arterial pressure, and right ventricular failure as found clinically [38]. However, the effects of either the high pressure or the VEGF receptor inhibition itself on proximal PA structure and function have not been documented. We do not think SU5416 has a direct effect on large PA remodeling because: 1) we have observed a normal endothelial layer (e.g. intact intima and no intimal thickening) in treated PAs by histology; 2) we did not find significant changes in cell proliferation or apoptosis in large PAs; and 3) we did not observe significant changes in systemic pressure with SU5416 treatment in additional groups of mice (not shown). This last finding is consistent with the reports of Ciuclan et al that there are no changes in systemic pressure due to either SU5416+hypoxia treatment or SU5416 alone [28]. Therefore, we conclude that the structural and functional changes observed in large PAs here are a result of chronic pulmonary pressure elevation.

Frequency-dependent viscoelastic behavior in large arteries (e.g. carotid, aorta, etc.) has been reported in previous studies. However, typically these studies were performed with healthy vessels, from large animals or humans. The present study is the first to investigate the viscoelastic properties of mouse arteries in both healthy and hypertensive conditions. From the pressure-stretch hysteresis loops, we observed very different frequency-dependent behaviors in healthy and hypertensive PAs (Fig 5A). The loop shape changed from a narrow, more horizontally-oriented and asymmetric ellipse (similar to the classic “J” profile of the loading curve obtained in static mechanical tests [39]) to a wider, more vertically-oriented and symmetric ellipse as frequency increased in control PAs. Such behavior has not been reported before and we do not know if this is specific to pulmonary arteries or a universal characteristic of conduit arteries. Moreover, in control PAs, as the frequency increased, the maximal stretch remained the same but the minimal stretch increased. The same behavior remained in the stress-strain hysteresis loops (data not shown). It is known that elastin is loaded at low strain whereas collagen is recruited at high strain as mechanical loading increases[40]. Therefore, we anticipate the increased minimal strain would lead to an earlier recruitment of collagen fibers [25], [41]and subsequent increase in arterial stiffness since collagen is stiffer than elastin. This may explain why the normalized stiffness κ (κ/κ_ CTL, 0.01 Hz_) and normalized elastic modulus E (E/E_ CTL, 0.01 Hz_) increased as frequency increased (Fig. 6A and B) in control PAs.

On the other hand, in hypertensive PAs, the maximal and minimal strain of each loop were both higher than those of control PAs and they remained fairly constant as frequency increased (Fig. 5B). This may contribute to the different frequency-dependent behavior in κ/κ_ CTL, 0.01 Hz_ and E/E_CTL, 0.01 Hz_ in 21d HySu group (Fig. 6A and B), which will be discussed in the next paragraphs. In addition, note that the entire stretch range in hypertensive PAs was ∼1.2–1.5, whereas it in the normoxia PAs was ∼1.2–2 (Fig. 5), which suggests an overall stiffening of the artery (decreased stretch for comparable pressurization) even at the lowest testing frequency. We have shown before that large PA remodeling in response to mild PAH leads to an early recruitment of collagen [25], [41] but to what extent the collagen is recruited is unknown. It is likely that in the hypertensive PAs, a higher percentage of collagen fibers are engaged and load-bearing than in normal PAs at the same pressure.

Our measurements of dynamic elastic modulus ratio (E/E_ CTL, 0.01 Hz_) demonstrate constant modulus ratios in both control and hypertensive PAs over the frequency range 0.01 – 10 Hz. The similar dynamic material property at a physiological frequency between control and hypertensive PAs seems counterintuitive because there was increased collagen and proteoglycan in hypertensive PAs and we also observed a significant increase in static elastic modulus in these PAs. However, it has been reported before that in rat carotids, hypertension decreases compliance and distensibility when measured in static tests, but has no significant effect when measured under dynamic conditions (i.e., in response to pulsatile pressure) [42]. In addition, the rat carotid artery wall was markedly stiffer in response to pulsatile pressure compared to static pressure differences over the same range [42]. Here, we also observed much higher dynamic E than E measured from static conditions in both control and hypertensive PAs. These findings show that arterial mechanical behavior changes significantly from static to dynamic conditions. These differences may be due to different engagement or interactions between ECM proteins in cyclic loading, and our results suggest collagen and proteoglycans are involved, but the mechanisms remain unknown. Since arteries are under pulsatile pressure in vivo, these dynamic structure-and-function relationships are highly clinically relevant.

At frequencies well above the normal heart rate, the modulus ratio increased in control PAs and decreased in hypertensive PAs; consequently, the modulus ratio became larger in control PAs compared to the hypertensive PAs at these super-physiological frequencies (p<0.05). For the control PAs, we speculate that the relationship between modulus ratio and frequency is sigmoidal with the inflection point determined in part by the normal physiological heart rate. That is, in prior studies on canine and human large arteries in which the normal heart rate is ∼1 Hz, the ratio of dynamic elastic modulus to static or quasi-static modulus increases rapidly at 2- to 3-times the natural heart rate and then plateaus at higher frequencies (10- to 20-times higher than the natural heart rate) [10], [11], [13], [43]. In our studies on mouse PAs, we also observed a rapid increase in modulus ratio just above the natural heart rate (∼10 Hz) but were unable to test at 10-fold higher frequencies at which a plateau may have occurred.

The different frequency-dependent behavior in dynamic structural (κ) and material (E) stiffness in hypertensive PAs is a novel finding of this study. In linearly viscoelastic, passive materials, stiffness increases with frequency[44]. But it is clear from the hysteresis loops (Fig. 5) that the PAs behave as nonlinearly viscoelastic materials, which do not necessarily follow the same trends as linear viscoelastic materials. As shown in Fig. 5, the changes in hysteresis loop shape with frequency in hypertensive PAs were markedly different from the control PAs. We have ruled out the possibility that the testing system is approaching resonance frequency by silicone tube tests; therefore, the hysteresis loop behavior is a reflection of the diseased artery mechanical properties. We attribute the ‘abnormal’ frequency-dependent decrease in stiffness to the potential different organization and function of the ECM and cell-matrix interactions in the diseased artery wall. Whether the frequency-dependence is modulated by the ECM interactions, SMCs in a passive state or cell-matrix interactions requires further investigation. Since there was a trend that the collagen/proteoglycan ratio was decreased in the 21d HySu PAs (7±2 vs. 3±0.5, p = 0.06), we suspect that the relative increase of proteoglycan may play a role in this behavior.

Changes in SMC activity by treatment with vasoconstrictors or vasodilators are known to alter energy loss in conduit arteries [16], [45], [46]. In tissues without muscle cells, such as tendon and cartilage, collagen and proteoglycans are known to affect energy loss [47]–[51]. However, little is known about whether these ECM proteins affect arterial viscoelasticity and, if they do, if the resulting viscoelastic properties are frequency-dependent. In the present study, we have obtained novel findings on the viscous behavior of both healthy and hypertensive PAs in the absence of any SMC tone. In particular, we observed an increase in damping capacity (D) as frequency increased for both healthy and hypertensive PAs. Such viscous behavior has been reported in human ligament[52] and may be related to the inherent viscoelasticity of ECM proteins where, for example, the covalent cross-linking of collagen has been found to be correlated to the PA viscoelasticity[53]. Moreover, the D at high frequencies (>1 Hz, including the normal mouse heart rate of ∼10 Hz) was lower in 21d HySu PAs compared to the control PAs. This difference is likely due to the accumulation of collagen and proteoglycans with PAH, and perhaps their ratio, as we did not observe other significant biological changes.

In this study, we did not measure arterial viscoelasticity with SMC activity for two reasons: first, it is technically impossible to perform serial cyclic inflation tests on whole arteries at different frequencies with the same degree of SMC contraction. We have previously observed a significant loss of maximal contraction (as induced by the potent vasoconstrictor U46619) after the first cycle of pressurization at 0.01 Hz. Second, unlike in systemic arteries, in conduit PAs, the SMC passive state is nearly indistinguishable from the normal SMC tone state. Our group has previously found similar diameter and mechanical properties of proximal PAs in a passive state (as induced by the vasodilators sodium nitroprusside and the rho-kinase inhibitor Y27632 or calcium-free medium) as compared to a normal tone state [24], [41]. Similar conclusions have been reported in the literature of pulmonary hypertension using other animal models [31], [54]–[56]. Thus, we expect our results in a passive SMC state to hold true in vivo, in a normal SMC tone state.

To identify the possible biological factors responsible for the mechanical property changes, we examined cellular and extracellular changes in the PAs. We did not observe significant changes in the rate of vascular cell proliferation or apoptosis, which is consistent with previous findings in large PAs in pulmonary hypertension[28], [31]. Also, we have shown before that with the progression of mild PAH, mouse large PAs accumulate collagen with minimal changes in elastin[24]. Similar observations were found in the 21d HySu PAs: there was increased total collagen thickness (Fig 2) but no significant changes in elastin. We speculate the collagen accumulation is mainly responsible for the increases in PA structural stiffness and static elastic modulus. We further studied another category of ECM molecules – proteoglycans, which are glycosylated proteins that provide hydration and swelling pressure to the tissue to enable it to withstand compressional forces. There is strong prior evidence that proteoglycans play a role in tissue viscoelastic properties [47]–[49]. For example, decorin, a member of proteoglycan family, is known to regulate collagen fibril formation[57] and has been shown to affect viscoelasticity in mouse tendon[58]. Interestingly, here we observed an increase in both percentage and total content of proteoglycan in 21d HySu PAs (Fig 3). We did not investigate the independent effects of proteoglycan and collagen on PA viscoelasticity in the present study, but our results suggest that both molecules may be involved. The concomitant changes in these ECM proteins and arterial viscoelasticity, and lack of changes in SMC proliferation and apoptosis, coupled with the absence of SMC tone (achieved pharmacologically) suggest that ECM proteins are critical to arterial viscoelasticity in a way that has not been previously described.

The impact of conduit arterial viscoelastic properties on ventricular function has not been fully investigated but may be relevant to pathological conditions where ventricular function is impaired[28]. For instance, the effect of arterial stiffening (i.e. decreased elasticity) is well known to contribute to the ventricular afterload and decoupling of the RV from its pulmonary vasculature [59]–[61]. Increased stiffness may increase wave reflections, which in turn augment the RV afterload [62]–[64]. The dampening function of conduit arteries achieved through viscosity is beneficial in normal physiological conditions because it absorbs the energy from the wave reflections. In sheep with acute pulmonary hypertension, in the isobaric condition, SMC activation-mediated increase in PA energy loss led to a decrease in wave reflections and increase in characteristic impedance, thus reducing the fraction of oscillatory to total RV hydraulic power and improving hemodynamic function[18]. These authors did not calculate the damping ratio but we estimate it was increased in the SMC activation group. An increase in conduit arterial viscosity or energy loss has also been reported in systemic and pulmonary hypertensive patients[16], [45], [65]. However, because these measurements were obtained under different in vivo pressures (i.e. higher pressures for hypertensive subjects and normal pressures for control subjects), the damping ratio calculated as dissipated energy/total energy may not necessarily increase due to the simultaneous increase in total energy[16]. In our study, the hypertensive PAs had a 41% decrease in damping capacity at the physiological heart rate (∼10 Hz). Such marked reduction in the cushion function may significantly increase flow pulsatility to downstream PAs, which is detrimental to distal arterial remodeling and stiffening [66]. Overall, whether the combined mechanical changes in proximal conduit PAs and distal small PAs may lead to an increase, decrease or preservation of the wave reflections, which will then affect the RV afterload, remains an open question and needs further investigation.

## Conclusion

In summary, we present for the first time the frequency-dependent viscoelastic properties of control and hypertensive mouse extralobar pulmonary arteries. Frequency-dependent changes in arterial stiffness, elastic modulus and damping capacity were found in the absence of SMC changes and in the presence of collagen and proteoglycans accumulation, which suggests a new role of ECM proteins in arterial viscoelasticity in PAH. The viscoelastic changes of proximal pulmonary artery may affect right ventricular afterload, thereby contributing to right ventricular failure in severe PAH.
